# Validity of claims-based definition of number of remaining teeth in Japan: Results from the Longevity Improvement and Fair Evidence Study

**DOI:** 10.1371/journal.pone.0299849

**Published:** 2024-05-07

**Authors:** Yudai Tamada, Taro Kusama, Sachiko Ono, Megumi Maeda, Fumiko Murata, Ken Osaka, Haruhisa Fukuda, Kenji Takeuchi

**Affiliations:** 1 Department of International and Community Oral Health, Tohoku University Graduate School of Dentistry, Sendai, Miyagi, Japan; 2 Department of Preventive Medicine, Nagoya University Graduate School of Medicine, Nagoya, Aichi, Japan; 3 Division of Statistics and Data Science, Liaison Center for Innovative Dentistry, Tohoku University Graduate School of Dentistry, Sendai, Miyagi, Japan; 4 Department of Eat-loss Medicine, Graduate School of Medicine, The University of Tokyo, Tokyo, Japan; 5 Department of Health Care Administration and Management, Kyushu University Graduate School of Medical Sciences, Fukuoka, Japan; Far Eastern Memorial Hospital, TAIWAN

## Abstract

**Background:**

Secondary healthcare data use has been increasing in the dental research field. The validity of the number of remaining teeth assessed from Japanese dental claims data has been reported in several studies, but has not been tested in the general population in Japan.

**Objectives:**

To evaluate the validity of the number of remaining teeth assessed from Japanese dental claims data and assess its predictability against subsequent health deterioration.

**Methods:**

We used the claims data of residents of a municipality that implemented oral health screening programs. Using the number of teeth in the screening records as the reference standard, we assessed the validity of the claims-based number of teeth by calculating the mean differences. In addition, we assessed the association between the claims-based number of teeth and pneumococcal disease (PD) or Alzheimer’s disease (AD) in adults aged ≥65 years using Cox proportional hazards analyses.

**Results:**

Of the 10,154 participants, the mean number of teeth assessed from the claims data was 20.9, that in the screening records was 20.5, and their mean difference was 0.5. During the 3-year follow-up, PD or AD onset was observed in 10.4% (3,212/30,838) and 5.3% (1,589/30,207) of participants, respectively. Compared with participants with ≥20 teeth, those with 1–9 teeth had a 1.29 (95% confidence interval [CI]: 1.17–1.43) or 1.19 (95% CI: 1.04–1.36) times higher risk of developing PD or AD, respectively.

**Conclusion:**

High validity of the claims-based number of teeth was observed. In addition, the claims-based number of teeth was associated with the risk of PD and AD.

## Background

Secondary use of healthcare data (e.g., administrative claims databases) has been increasing in the field of dental research [1, 2]. Such health data are often large and population-representative, and the results of their analyses are expected to provide information on therapeutic effectiveness and safety in real-world settings. However, because such data were initially collected for non-research purposes, it is possible that they do not always precisely reflect patients’ health conditions. For instance, claims data are collected for reimbursement purposes; hence, if patients have a record of a disease, it is sometimes difficult to differentiate whether the disease was registered because the patient had the disease or because the healthcare providers wanted to provide disease-related procedures (e.g., the patients who required a chest scan might be recorded as having lung cancer, in extreme cases). Therefore, validation studies are required to minimize misclassification and enhance the reliability of secondary health data analyses [3].

In Japan, various oral health promotion policies, such as the 8020 Campaign [4] and Health Japan 21 [5], have been implemented to maintain citizens’ oral health and prevent tooth loss. From a policymaking perspective, it is important to evaluate the effectiveness of such policies by assessing whether people retained their teeth after policy implementation. In this process, the number of remaining teeth needs to be assessed accurately and in a timely manner; however, self-reported questionnaires or dental examinations, which take time and are costly, are often used. Although there is potential for the number of teeth to be efficiently assessed using claims data, the use of claims-based assessment is limited, perhaps because its validity has not been sufficiently established. Several studies have reported the validity of the number of remaining teeth in dental claims data [6, 7]; however, these studies were limited to patients in one hospital [6] or to assessments at the population level [7], rather than at the individual level. Therefore, it remains unclear whether the number of remaining teeth in dental claims data is valid for the general population if assessed using individual-level data.

Tooth loss is one of the most prevalent conditions affecting millions of people worldwide [8] and is associated with a higher risk of mortality [9] and systemic diseases [10–14]. Although various systemic diseases have been reported to be associated with oral diseases, a recent umbrella review indicated that there is a greater amount of strong evidence for an association between tooth loss and neurodegenerative diseases, such as Alzheimer’s disease (AD), than other diseases [15]. Another review concluded that periodontitis, the main cause of tooth loss [16], is a strong risk factor for pneumonia [17]. Given that pneumococcal pneumonia, a lung infection caused by *Streptococcus pneumoniae*, is the most common type of pneumonia [18], tooth loss is also considered to be associated with pneumococcal disease (PD). However, no study has investigated the association between the number of teeth and PD or AD using Japanese claims data.

In this context, the primary purpose of this study was to examine the validity of the claims-based number of remaining teeth assessed using Japanese dental claims data from community-dwelling adults using oral health screening records as reference standards. As the secondary purpose, this study aimed to assess whether the claims-based number of teeth predicts the subsequent risk of PD and AD in adults aged ≥65 years, using the Japanese claims data.

## Methods

### Study population and setting

This study used data from the Longevity Improvement & Fair Evidence (LIFE) Study [19], a longitudinal multi-region community-based database project that aims to provide evidence for extending healthy life expectancy and reducing health disparities. We used data from a municipality with a residential population of approximately 700,000 that provided oral health screening data and healthcare claims data for two insurance systems (i.e., the National Health Insurance and the Latter-Stage Older Persons Health Care System). The municipality has implemented two annual oral health-screening programs (i.e., periodontal disease screening and oral healthcare screening). Periodontal disease screening was conducted for adults aged 20–70 years in five-year increments from June to November, and oral healthcare screening was conducted for those aged ≥65 years throughout the year. Both screenings were conducted in designated dental clinics as individual screenings, and the dentists determined the number of remaining teeth. National Health Insurance is a public insurance scheme for those not employed; enrollees include self-employed or part-time workers, retired workers, and their dependents. In addition, the Latter-Stage Older Persons Health Care System is a public insurance scheme for those aged ≥75 years; the enrollees cover all the residents aged ≥75 years in the municipality. The health insurance system in Japan has been described in detail elsewhere [20]. In the LIFE Study, a unique research ID was assigned to each resident by the data managers, and each data point was linked at the individual level.

**Fig 1:**
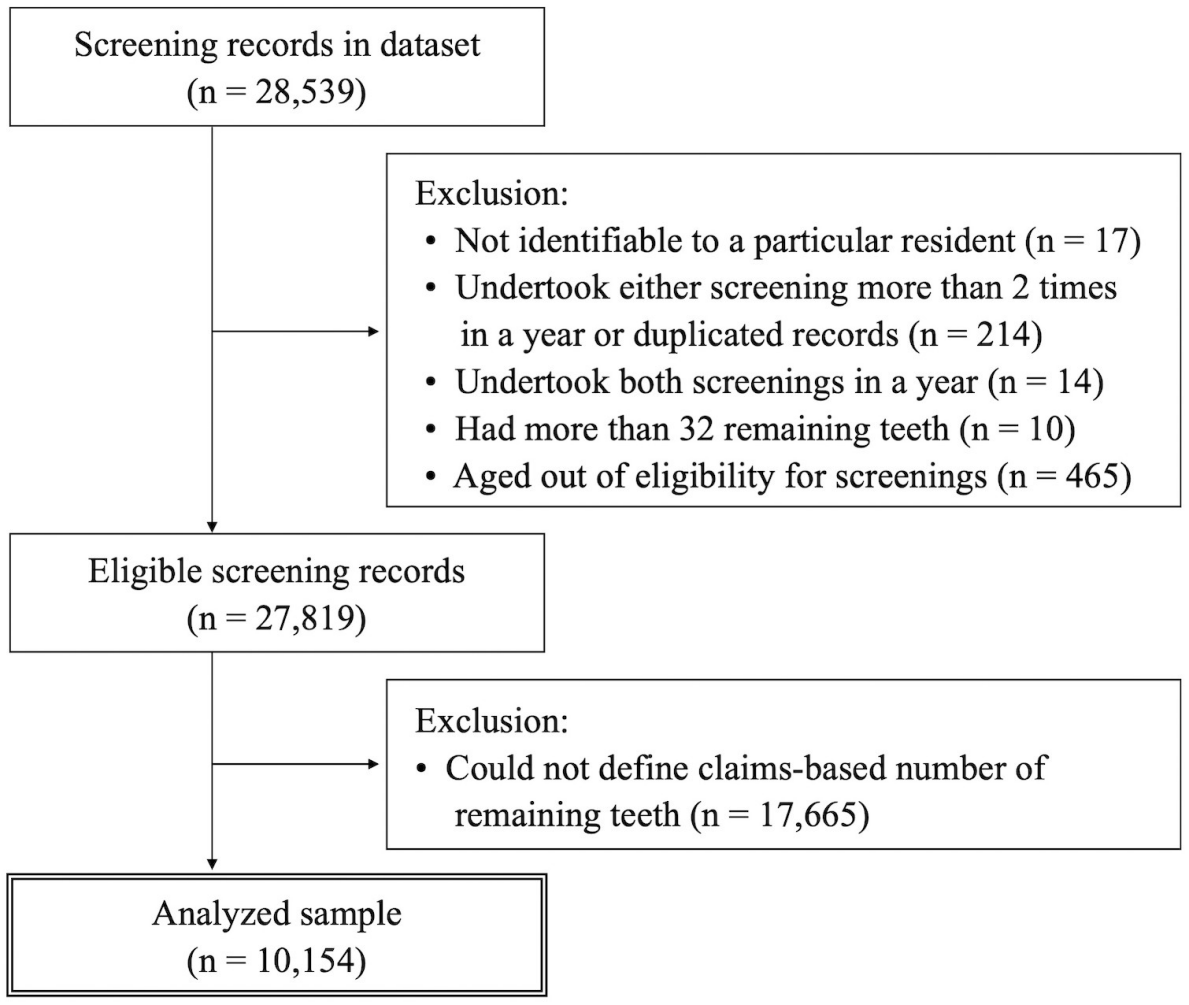
Diagram of analytical sample selection.

Two series of analyses were performed. First, we examined the validity of the claims-based number of remaining teeth, using information on the number of teeth, based on oral health screening records as the reference standard (validation analysis). The number of teeth in the screening records was used as the reference standard because it was expected to be more reliable than our algorithm-based estimation as it was determined by dentists following an instruction manual [21]. We extracted 28,539 screening records from the database that were collected in the fiscal years 2018 or 2019 (April 2018–March 2020). We excluded the following screening records: records that were not identifiable to a particular resident (n = 17), those of people who undertook either screening (periodontal disease screening or oral healthcare screening) ≥2 times in a year or duplicated records (n = 214), those of people who undertook both screenings in a year (n = 14), those recorded as having ≥32 teeth (n = 10), and those of people not in the eligible age range for the screenings (n = 465). Of the 27,819 eligible records, 10,154 could be linked with claims-based data on the number of remaining teeth (Fig 1). In the main analysis, the participants’ claims-based number of teeth was assessed using their 12 months of dental claims data prior to the screening month. In the sensitivity analysis, we defined the claims-based number of teeth using the participants’ 12 months of dental claims data after the screening month. Second, we examined the associations between the claims-based number of teeth and the risks of PD or AD, which are previously reported consequences of poor oral health (prediction analysis) [10–14]. Using dental claims between April 2016 and March 2017, we assessed the number of teeth of eligible participants, aged ≥65 years as of March 2017, who had continuous health insurance enrollment during the assessment period. After excluding participants diagnosed with PD or AD during the number-of-teeth assessment period, we followed the participants from April 1, 2017, to March 31, 2020. The design diagram [22] is shown in S1 Fig.

### Claims-based assessment of number of remaining teeth

Although direct information on the number of remaining teeth is not included in the Japanese dental claims data, monthly data on the number of treated teeth per treatment are available using the listed dental formulas. The dental formula is a six-digit code, with the first four digits identifying the teeth (e.g., the maxillary central incisor), the next digit indicating the status of the teeth (e.g., remaining or missing), and the last digit assigned to the treated part of the teeth (e.g., mesial or distal). Considering that all remaining teeth would be assessed in basic/comprehensive periodontal examinations, we extracted the dental claims for the months when the procedures were conducted by identifying them with the corresponding procedure codes (304000410, 304000510, 304000610, 304000710, 304000810, and 304000910). After excluding the dental formulas of missing teeth identified by the fifth digit, we counted the number of dental formulas per treatment. Finally, the maximum number of dental formulas in the assessment period (12 months) was defined as the participants’ claims-based number of remaining teeth. In addition, in the sensitivity analysis, we defined the number in the latest claims data in the assessment period as the claims-based number of remaining teeth.

### PD and AD assessments

In this study, according to definitions used in previous studies [23, 24], the onset of PD was identified using the original Japanese diagnostic codes in the medical claims data (S1 Table). As the claims included only monthly data on diseases, we defined the onset dates in two ways, based on the types of patient care: (i) for outpatients, the 15th of the month when they were diagnosed with PD for the first time during follow-up, and (ii) for inpatients, the admission date for in-hospital PD treatments for the first time during follow-up. The onset of AD was identified using the International Classification of Diseases, 10th Revision (ICD-10) code (G30) [25, 26], and the onset dates were defined in the same manner as those for PD. Additionally, we conducted a sensitivity analysis to identify the onset of AD using a combination of the corresponding ICD-10 code and prescriptions for AD medications (donepezil, rivastigmine, galantamine, and memantine). In the analysis, the onset date was defined as the first prescription date of AD medication during follow-up.

### Statistical analysis

In the validation analysis, we generated a Bland–Altman plot and heatmap and calculated the intraclass correlation coefficient (ICC) between the claims-based number of teeth and the number of teeth in the screening records. In addition, we calculated ICCs based on the number of teeth groups and age groups. For sensitivity analyses, we generated a Bland–Altman plot using the claims-based number of teeth defined by the participants’ 12 months of dental claims data after the screening month. In addition, we generated heatmaps using the subsample stratified by age group (20–39/40–64/65–74/≥75 years) to assess the consistency of validity of the claims-based number of teeth across life stages. The age-group classification was employed to approximately capture the participants’ life stages as young (20–39 years), middle-aged (40–64 years), early older (65–74 years), and late older adults (≥75 years).

In the prediction analysis, log-rank tests were used to compare the cumulative incidence curves by the claims-based number of teeth categories (1–9/10–19/≥20 teeth), at baseline. In addition, Cox proportional hazards models were used to estimate the hazard ratios (HRs) for the onset of PD/AD during the 3-year follow-up period, according to the claims-based number of teeth categories, after adjusting for sex, age group, hypertension (ICD-10 code: I10), and diabetes (ICD-10 codes: E10–E14). Their 95% confidence intervals (CIs) were obtained by bootstrapping with 1,000 replications [27]. The proportional hazard assumption was verified using Schoenfeld residuals. Follow-up was censored at the onset of PD/AD, disenrollment from health insurance, or end of follow-up (March 31, 2020), whichever occurred first. In the additional analysis, we used a composite variable defined by combining the claims-based number of teeth and denture use status (1–9 teeth not using dentures/1–9 teeth using dentures/10–19 teeth not using dentures/10–19 teeth using dentures/≥20 teeth) as the exposure instead of the claims-based number of teeth. Denture use status was assessed by the routinely reimbursed denture management fee (procedure codes: 308002510, 308002610, 308002710, or 308004210) appearing in the claims-based number of teeth assessment period.

All analyses were conducted using Stata (version 17.0; Stata Corp., College Station, TX, USA). This study followed the Strengthening the Reporting of Observational Studies in Epidemiology (STROBE) guidelines. Data analysis was conducted from September 27, 2022, to June 27, 2023.

### Ethical considerations

This study was approved by the Kyushu University Institutional Review Board for Clinical Research (approval number: 22114–02) and the Ethics Committee of Tohoku University Graduate School of Dentistry (approval number: 23835). Data usage approval was obtained from the municipality’s Personal Information Protection Review Board. Informed consent was waived by the ethics committee since this study was based on previously collected medical records data. All the information that we had access were fully anonymized and could not be used to identify individual participants during or after data collection.

## Results

### Validity of claims-based number of remaining teeth

Table 1 presents the descriptive statistics of the analytical sample in the validation analysis according to the oral health screening programs. Among the 10,154 participants, 1,999 (age: 59.0±12.8 years [mean±standard deviation (SD)]) underwent periodontal disease screening and 8,155 (age: 77.1±6.0 years [mean±SD]) underwent oral healthcare screening. Of the participants from the periodontal disease screening group, the mean number of teeth assessed from the claims data was 24.6 (SD: 4.9) and that in the screening records was 24.7 (SD: 5.2). However, among those from the oral healthcare screening group, the mean number of teeth assessed from the claims data was 20.1 (SD: 7.2) and that in the screening records was 19.5 (SD: 7.7).

**Table 1:** Characteristics of analytical sample according to oral health screening programs.

	Total (n = 10,154)	Periodontal disease screening (n = 1,999)	Oral healthcare screening (n = 8,155)
Number of teeth in screening records			
mean (SD)	20.5 (7.6)	24.7 (5.2)	19.5 (7.7)
median (IQR)	23 (16–26)	26 (23–28)	22 (14–26)
Claims-based number of teeth			
mean (SD)	20.9 (7.0)	24.6 (4.9)	20.1 (7.2)
median (IQR)	23 (17–27)	26 (23–28)	22 (15–26)
Sex, n (%)			
Male	3,917 (38.6)	731 (36.6)	3,186 (39.1)
Female	6,237 (61.4)	1,268 (63.4)	4,969 (60.9)
Age^†^			
mean (SD)	74.6 (10.6)	59.0 (12.8)	77.1 (6.0)
median (IQR)	75 (70–80)	65 (50–70)	77 (73–81)
Age group, n (%)			
20–39 years (20, 25, 30, or 35 years)	164 (1.6)	164 (8.2)	—
40–64 years (40, 45, 50, 55, or 60 years)	731 (7.2)	731 (36.6)	—
65–74 years	3,799 (37.4)	1,104 (55.2)	2,695 (33.1)
≥75 years	5,460 (53.8)	—	5,460 (67.0)

Abbreviations: SD = standard deviation; IQR = interquartile range.

^†^ Age in a fiscal year (e.g., participants born in 1958 were regarded as 60 years old in fiscal year 2018).

**Fig 2:**
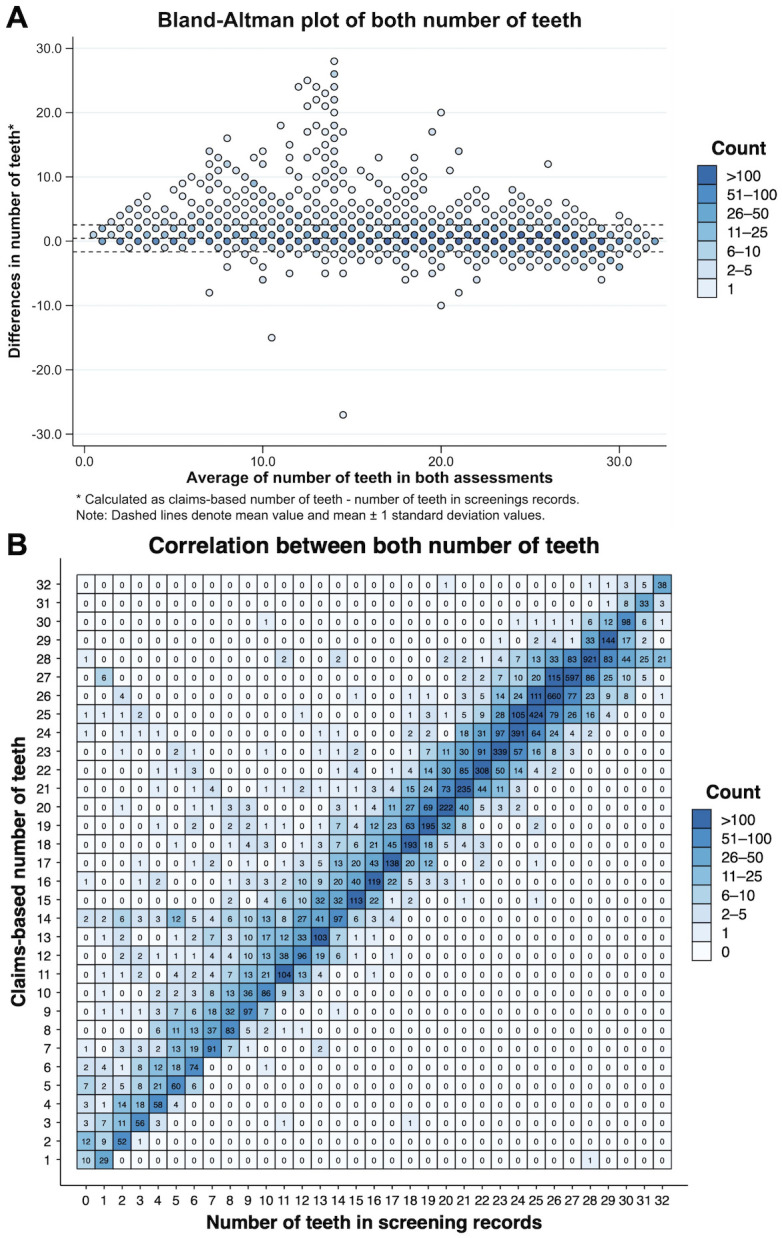
(A) Bland-Altman plot of claims-based number of teeth and number of teeth in screening records. (B) Heatmap of claims-based number of teeth and number of teeth in screening records.

Fig 2A is the Bland–Altman plot of the number of teeth between the claims-based assessment and the screening records. Overall, the number of teeth assessed from the claims data was slightly higher than that in the screening records and their mean differences were 0.5 (SD: 2.1) in all participants, 2.2 (SD: 4.3) in those with 1–9 teeth, 0.7 (SD: 1.8) in those with 10–19 teeth, and 0.0 (SD: 1.1) in those with ≥20 teeth. In addition, the mean difference between the number of teeth in the screening records and that defined using the participants’ dental claims data after the screening month was 0.2 (SD: 2.1) (S2 Fig). Fig 2B shows the heatmap of the number of teeth assessed from the claims data and that in the oral health screening records. The claims-based number of teeth was highly correlated with the number of teeth in the screening records (ICC: 0.96). In the stratified analysis based on age group, a higher correlation was observed in the older population (ICC: 20–39 years, 0.58; 40–64 years, 0.97; 65–74 years, 0.98; ≥75 years, 0.97) (S2 Table and S3 Fig). In addition, in the stratified analysis based on number of teeth group in screening records, a higher correlation was observed in the participants with a higher number of teeth (ICC: ≤9 teeth, 0.44; 10–19 teeth, 0.89; ≥20 teeth, 0.96) (S1 Table). Furthermore, similar results were observed in the sensitivity analysis that defined the claims-based number of teeth using the latest claims data in the 12 months before the screening month (S4 Fig). The mean differences between the claims-based assessment and the screening records were 0.3 (SD: 1.8), in all participants, 1.7 (SD: 3.6) in those with 1–9 teeth, 0.5 (SD: 1.7) in those with 10–19 teeth, and -0.1 (SD: 1.1) in those with ≥20 teeth.

### Association between claims-based number of teeth and PD/AD

**Fig 3:**
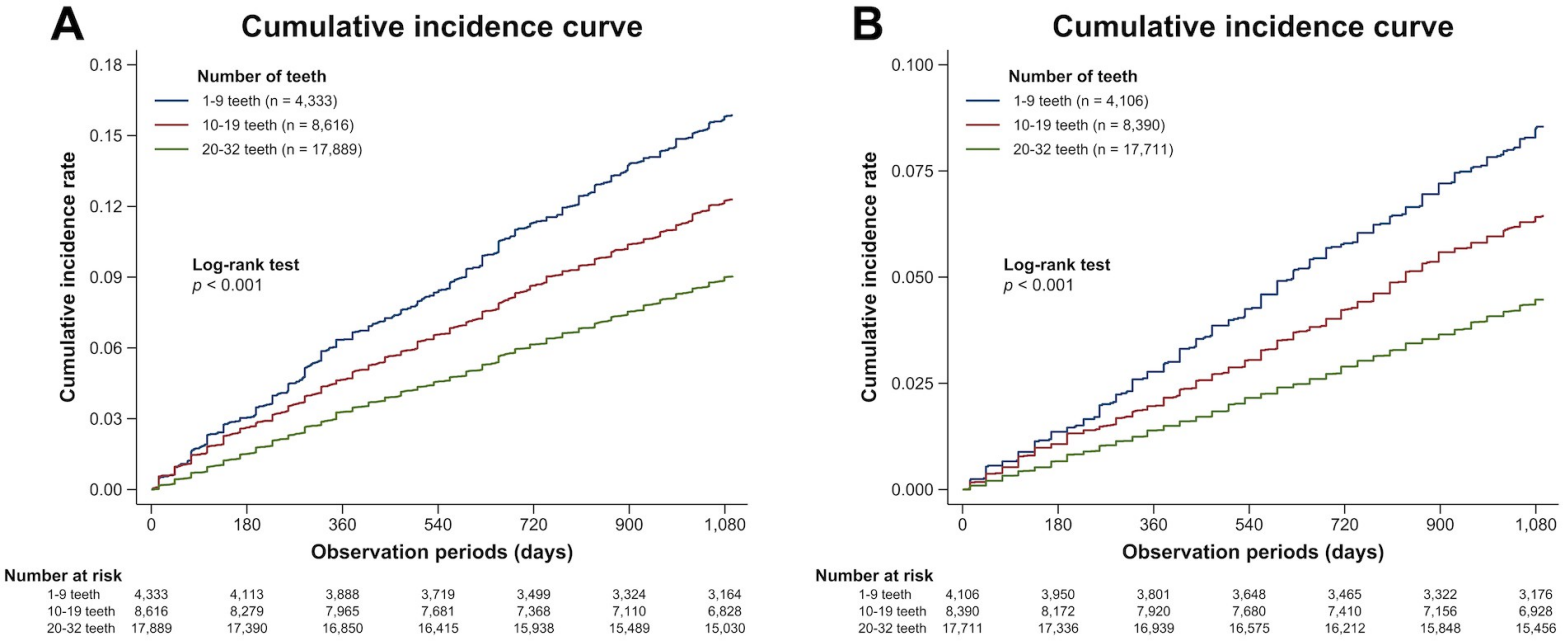
Cumulative incidence curves of (A) pneumococcal disease and (B) Alzheimer’s disease according to the claims-based number of remaining teeth.

S3 Table shows the descriptive statistics of the analytical sample used in the prediction analysis for PD, according to the number of teeth. Of the 30,838 participants (age: 76.4±7.3 years [mean±SD]), 14.1% had 1–9 teeth, 27.9% had 10–19 teeth, and 58.0% had ≥20 teeth. By the end of the follow-up period (mean: 2.7 years), PD onset was observed in 3,212 (10.4%) participants, especially those with a lower number of teeth, who were more likely to develop PD (log-rank test: *P*<0.001, Fig 3A). Descriptive statistics of the analytical sample and the cumulative incidence curves in the prediction analysis for AD are presented in S4 Table and Fig 3B. During the follow-up period (mean: 2.8 years), 1,589 (5.3%) participants developed AD; AD onset was more likely to be observed in participants with a lower number of teeth (log-rank test: *P*<0.001).

Table 2 shows the results of the Cox proportional hazards analyses of the associations between the claims-based number of teeth and PD/AD onset during follow-up. Even after adjusting for sex, age group, hypertension, and diabetes, fewer teeth remained associated with a higher risk of developing PD/AD. Compared with the participants with ≥20 teeth, those with 1–9 teeth had a 1.29 (95% CI: 1.17–1.42) times higher risk of PD and a 1.19 (95% CI: 1.04–1.35) times higher risk of AD. The results of the sensitivity analysis that integrated AD medications into the definition of AD, were similar to those of the primary analysis (S5 Table and S5 Fig). For instance, the participants with 1–9 teeth had a 1.08 (95% CI: 0.92–1.26) times higher risk of AD than those with ≥20 teeth. In the additional analysis that considered denture use status, participants using dentures had a lower risk of PD/AD than those not using dentures (S6 Table). For instance, the participants with 1–9 teeth using dentures had a 1.24 (95% CI: 1.12–1.38) times higher risk of PD and a 1.14 (95% CI: 0.98–1.32) times higher risk of AD than those with ≥20 teeth, while those with 1–9 teeth not using dentures had a 1.41 (95% CI: 1.21–1.66) times higher risk of PD and a 1.35 (95% CI: 1.08–1.70) times higher risk of AD.

**Table 2:** Association between the claims-based number of remaining teeth and the onsets of pneumococcal disease and Alzheimer’s disease during the follow-up.

	PD (n = 30,838)^†^			AD (n = 30,207)^†^		
	Person-years at risk	Crude incidence rate^‡^	HR (95% CI^§^)^¶^	Person-years at risk	Crude incidence rate^‡^	HR (95% CI^§^)^¶^
Number of teeth						
1–9 teeth	11,113.0	58.0	1.29 (1.17–1.42)	10,887.8	29.8	1.19 (1.04–1.35)
10–19 teeth	23,013.1	44.0	1.12 (1.04–1.21)	22,949.9	22.2	1.06 (0.94–1.18)
≥20 teeth	49,193.2	31.6	1.00 (Reference)	49,681.5	15.2	1.00 (Reference)

Abbreviations: PD = pneumococcal disease; AD = Alzheimer’s disease; HR = hazard ratio; CI = confidence interval.

^†^ Each outcome was separately included in the analytic models.

^‡^ Per 1,000 person-years.

^§^ Obtained by bootstrapping with 1,000 replications.

^¶^ Adjusted for sex, age group, hypertension, and diabetes.

## Discussion

In this study of community-dwelling adults in Japan, the claims-based number of remaining teeth was broadly consistent with that in the screening records. The difference in the number of teeth between claims-based assessment and screening records was slightly larger for participants with fewer teeth. In addition, we observed an inverse association between the claims-based number of teeth and risk of PD/AD onset among older adults, indicating that individuals with fewer teeth had a higher risk of PD or AD onset during the three-year follow-up period.

Our validation results for the claims-based number of remaining teeth were generally comparable with those previously reported [6, 7]. In a study of academic hospital patients, the mean difference was 1.0 teeth when the number of teeth assessed from the dental claims data was compared with that from radiographs [6], and this result was consistent with ours. In contrast, an ecological study based on claims-based assessment using a national database and summary results of a national survey in Japan [7] reported larger differences in the mean number of teeth among the older population. This difference to our findings may be partially explained by the tendency of the number of teeth in claims-based assessments to differ from the actual number of teeth in individuals with fewer teeth, such as older adults. We believe that our findings provide additional evidence that the claims-based number of teeth can be used as a parameter to determine the actual oral health status of community-dwelling populations.

The observed number of teeth in the claims-based assessment was slightly higher than that in the screening records, especially when participants had fewer teeth. A possible explanation is that we defined the claims-based number of teeth as the maximum number of candidates assessed using participants’ 12 months of claims data prior to the screening month. Hence, it is possible that if the participants had lost their teeth during the relevant 12-month period, the number of teeth in the screening records would have been fewer than in the claims-based assessment. This explanation is supported by our sensitivity analysis, which defined the claims-based number of teeth using participants’ claims data after the screening month. We found smaller differences in the number of teeth between the claims-based assessment and screening records than in the primary analysis.

Several possible mechanisms may underlie the association between a lower number of teeth and a higher risk of PD/AD. First, periodontal disease, which is the main cause of tooth loss [16], may induce systemic inflammation. Although this study did not include an evaluation of inflammation due to periodontal disease, the existing literature has demonstrated that chronic exposure to periodontal inflammation results in tooth loss [28, 29]. Hence, people with fewer teeth may have been continuously exposed to increased inflammatory mediators due to periodontal disease, at least until before losing their teeth, which is considered to contribute to the development of AD [30, 31]. Second, masticatory and swallowing dysfunctions caused by tooth loss [32, 33] may lead to subsequent functional decline and health behavioral changes that could be associated with diseases. For instance, swallowing difficulties increase the risk of aspiration of microbes from oral biofilms [34] and may contribute to the onset of PD. Active mastication stimulates cerebral blood flow and increases cerebral arousal [35]; hence, masticatory dysfunction can contribute to the onset of AD through a decline in brain function. Moreover, poor oral function is thought to decrease vegetable and fruit intake [36], and consequently increase the risk of cognitive impairment [37].

Our validation results for the claims-based number of teeth may have several implications. For research purposes, the claims-based number of teeth could be used as a covariate in a statistical model when conducting clinical epidemiology studies in which the number of teeth may be regarded as a confounding factor. In addition, the number of teeth is considered to be influenced by social and lifestyle-related factors throughout life [16, 38]. For instance, previous studies have indicated that social factors in childhood, such as adverse childhood experiences [39, 40], are associated with fewer remaining teeth, later in life. Given that the Japanese claims data do not include sufficient information on social background, the claims-based number of teeth may be used as a proxy that partially reflects the participants’ socioeconomic status and health conditions. For health policy evaluation purposes, the claims-based number of teeth can be used to assess the success of oral health promotion policies. Since claims data are already collected for reimbursement, municipal governments may be able to assess the oral health status of their residents by evaluating the number of teeth through claims-based assessments without costly primary data collection, such as dental examinations or surveys.

This study had several limitations. First, oral examinations in the screening programs were not performed by calibrated examiners; thus, the number of teeth in the screening records may have differed between examiners. However, considering that the examinations were performed following an instruction manual [21], misclassification may not have occurred often, which may not have significantly affected the results. Second, our analytical sample included only a limited number of younger participants, particularly those with fewer teeth. Therefore, our validation results should be interpreted with caution, especially when generalizing them to younger populations. Third, our analytical sample relied on a selective population of individuals who underwent periodontal disease screening or oral healthcare screening. However, there is little possibility that the input of dental formulas into the dental claims data was systemically different between people who underwent the screenings and those who did not. Therefore, we believe that our findings can be generalized. Fourth, we observed that the claims-based number of teeth was associated with PD/AD; however, there may be unadjusted potential confounders, such as education and income levels. Although there may be many potential confounders, we only adjusted for sex, age group, hypertension, and diabetes to assess whether the number of remaining teeth predicts subsequent PD/AD onset, rather than exploring their causality [41]. Hence, our results need to be interpreted with caution, as there may be no causal relationship. Fifth, if participants with cognitive impairment fail to maintain good oral hygiene and consequently lose their teeth, there may be a possibility of reverse causation in the association between the claims-based number of teeth and AD. However, by excluding participants who were diagnosed with AD during the 12 months before starting the follow-up from the analytical sample (S1 Fig), we can partially address the possibility of reverse causation.

In conclusion, our study demonstrated the validity of the claims-based number of remaining teeth in a community-dwelling population. In addition, a lower number of teeth were associated with a higher risk of PD/AD among older adults. Our findings may facilitate the use of dental claims data for research and health policy evaluation.

## Supporting information

S1 Fig Design diagram of this study. (PDF)

S2 Fig Bland-Altman plot of claims-based number of teeth and number of teeth in screening records using different definition of claims-based number of teeth. (PDF)

S3 Fig Heatmaps of claims-based number of teeth and number of teeth in screening records among participants aged (A) 20–39 years, (B) 40–64 years, (C) 65–74 years, or (D) ≥75 years. (PDF)

S4 Fig (A) Bland-Altman plot of claims-based number of teeth (latest one in assessment period) and number of teeth in screening records. (B) Heatmap of claims-based number of teeth (latest one in assessment period) and number of teeth in screening records.
(PDF)

S5 Fig Cumulative incidence curves of Alzheimer’s disease according to the claims-based number of remaining teeth using Alzheimer’s disease definition that integrates Alzheimer’s disease medications. (PDF)

S1 Table Definition of Japanese original diagnosis codes for pneumococcal disease. (PDF)

S2 Table Correlation between claims-based number of teeth and number of teeth in screening records by age group and number of teeth group in screening records. (PDF)

S3 Table Characteristics of analytical sample in prediction analysis for pneumococcal disease according to the claims-based number of remaining teeth. (PDF)

S4 Table Characteristics of analytical sample in prediction analysis for Alzheimer’s disease according to the claims-based number of remaining teeth. (PDF)

S5 Table Association between the claims-based number of remaining teeth and the onset of Alzheimer’s disease during follow-up using Alzheimer’s disease definition that integrates Alzheimer’s disease medications. (PDF)

S6 Table Association between the dental status and the onsets of pneumococcal disease and Alzheimer’s disease during the follow-up. (PDF)
