# Supplementary material for: Validity of claims-based definition of number of remaining teeth in Japan: Results from the Longevity Improvement and Fair Evidence Study
Source: PLoS One. 2024 May 7;19(5):e0299849. doi: 10.1371/journal.pone.0299849 (PMC11075880; doi:10.1371/journal.pone.0299849)
Supplement: S1 Fig — (PDF) [file pone.0299849.s001.pdf]

Figure S1. Design diagram of this study.

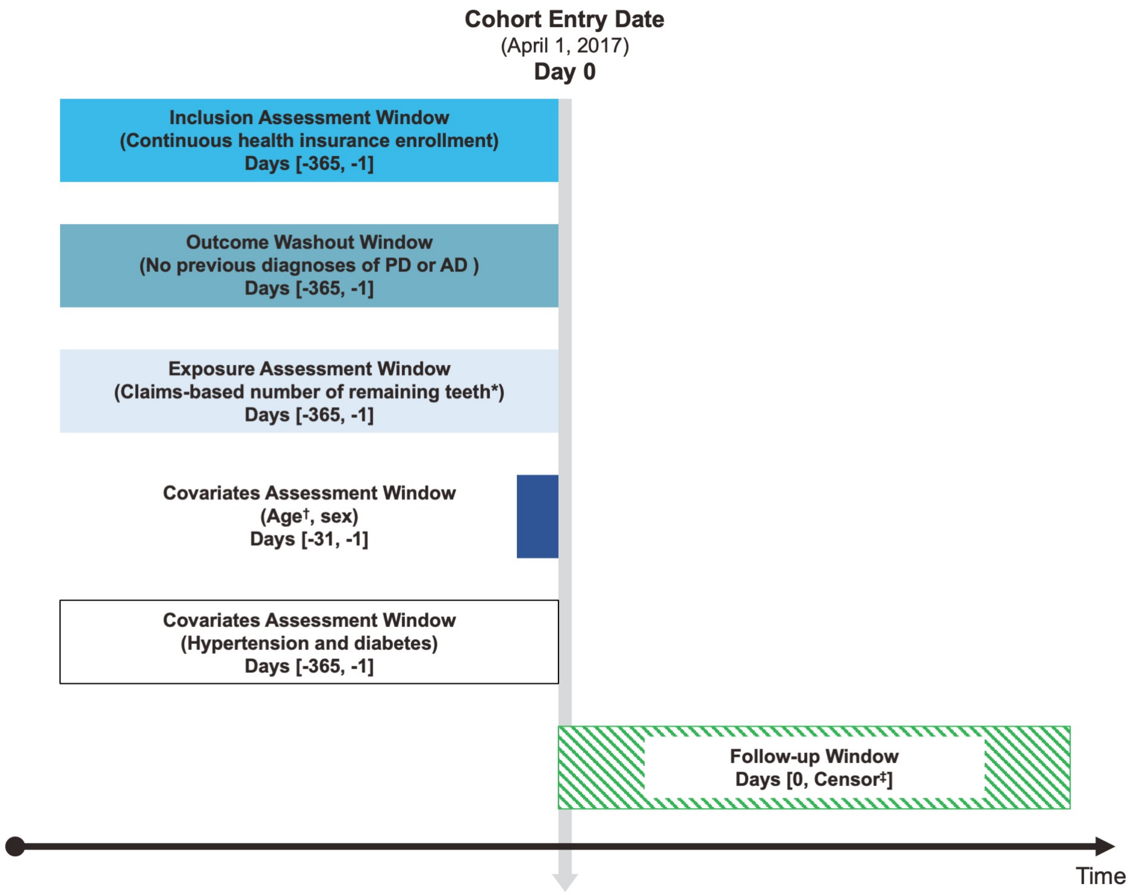

Abbreviations: PD = pneumococcal disease; AD = Alzheimer's disease.

* The maximum number in the candidates (dental formulas per treatment in dental claims for the months when basic/comprehensive periodontal examinations were conducted).

† Participants were regarded as they were born on 15th of their birth month because exact birthday are not available from the perspective the personal information protection. Age was as of the March 15, 2017.

‡ Earliest of the onset of PD/AD, disenrollment from health insurance, or end of follow-up (March 31, 2020).
