# Supplementary material for: Validity of claims-based definition of number of remaining teeth in Japan: Results from the Longevity Improvement and Fair Evidence Study
Source: PLoS One. 2024 May 7;19(5):e0299849. doi: 10.1371/journal.pone.0299849 (PMC11075880; doi:10.1371/journal.pone.0299849)
Supplement: S2 Fig — (PDF) [file pone.0299849.s002.pdf]

Figure S2. Bland-Altman plot of claims-based number of teeth and number of teeth in screening records using different definition of claims-based number of teeth.

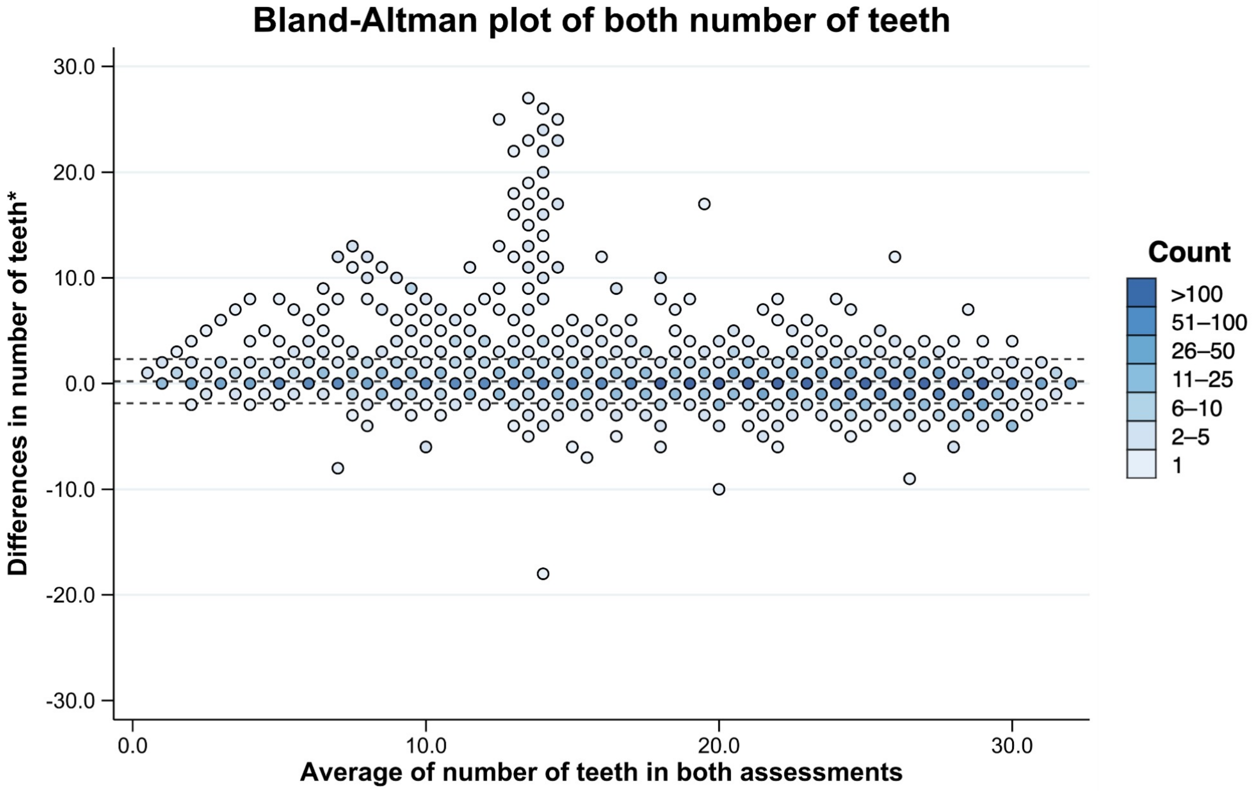

* Calculated as claims-based number of teeth - number of teeth in screenings records.
Note: Dashed lines denote mean value and mean ± 1 standard deviation values.

Note: The claims-based number of teeth was defined using the participants' 12 months of dental claims data after the screening month. We included only the oral health screenings data of the fiscal year 2018 because the claims data of until March 2020 were available.
