# Supplementary material for: Validity of claims-based definition of number of remaining teeth in Japan: Results from the Longevity Improvement and Fair Evidence Study
Source: PLoS One. 2024 May 7;19(5):e0299849. doi: 10.1371/journal.pone.0299849 (PMC11075880; doi:10.1371/journal.pone.0299849)

Figure S3. Heatmaps of claims-based number of teeth and number of teeth in screening records among participants aged (A) 20–39 years, (B) 40–64 years, (C) 65–74 years, or (D) ≥75 years.

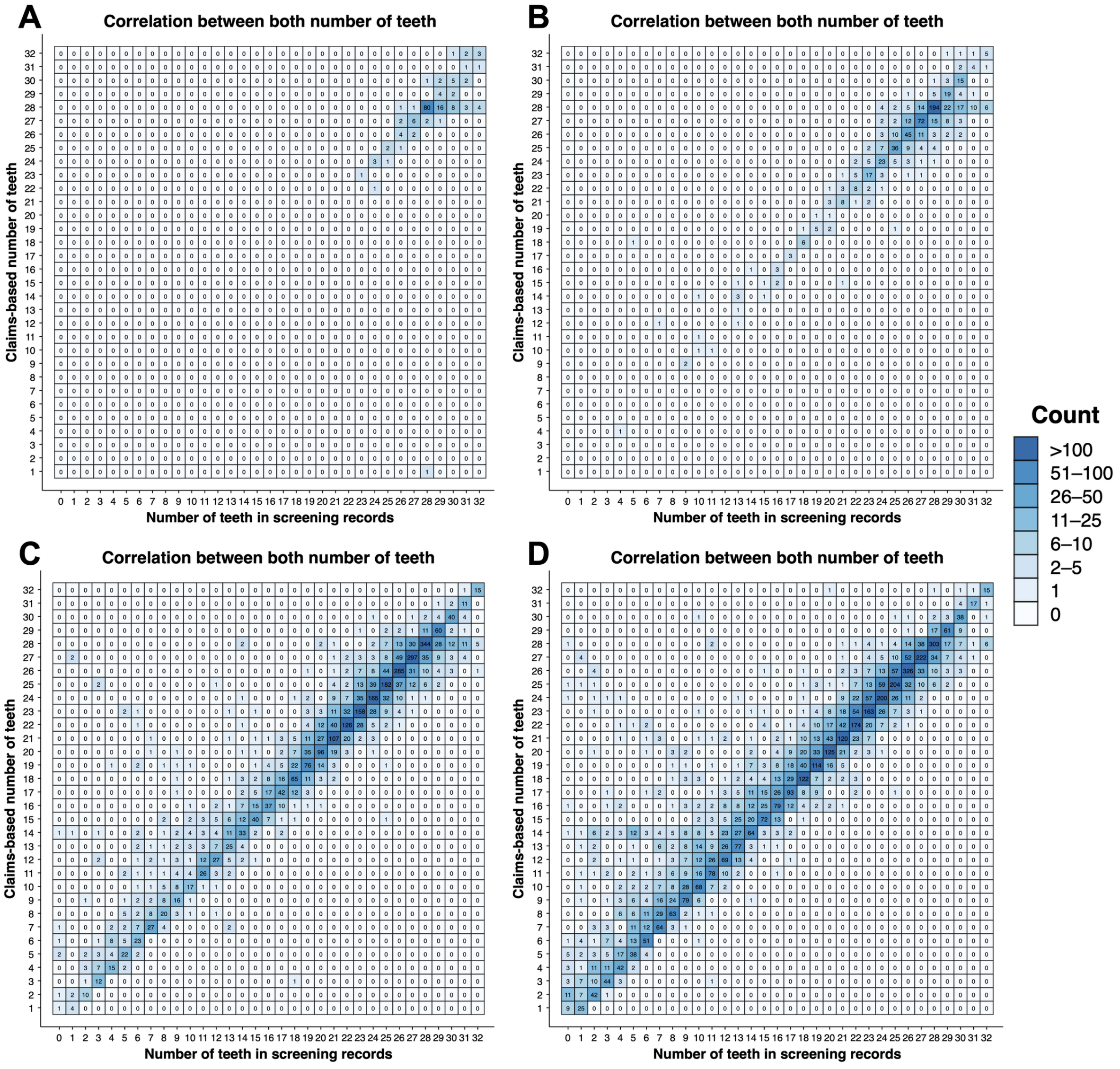

Supplement: S3 Fig — (PDF) [file pone.0299849.s003.pdf]
