# Supplementary material for: Validity of claims-based definition of number of remaining teeth in Japan: Results from the Longevity Improvement and Fair Evidence Study
Source: PLoS One. 2024 May 7;19(5):e0299849. doi: 10.1371/journal.pone.0299849 (PMC11075880; doi:10.1371/journal.pone.0299849)
Supplement: S4 Fig — (A) Bland-Altman plot of claims-based number of teeth (latest one in assessment period) and number of teeth in screening records. (B) Heatmap of claims-based number of teeth (latest one in assessment period) and number of teeth in screening records. (PDF) [file pone.0299849.s004.pdf]

Figure S4. (A) Bland-Altman plot of claims-based number of teeth (latest one in assessment period) and number of teeth in screening records. (B) Heatmap of claims-based number of teeth (latest one in assessment period) and number of teeth in screening records.

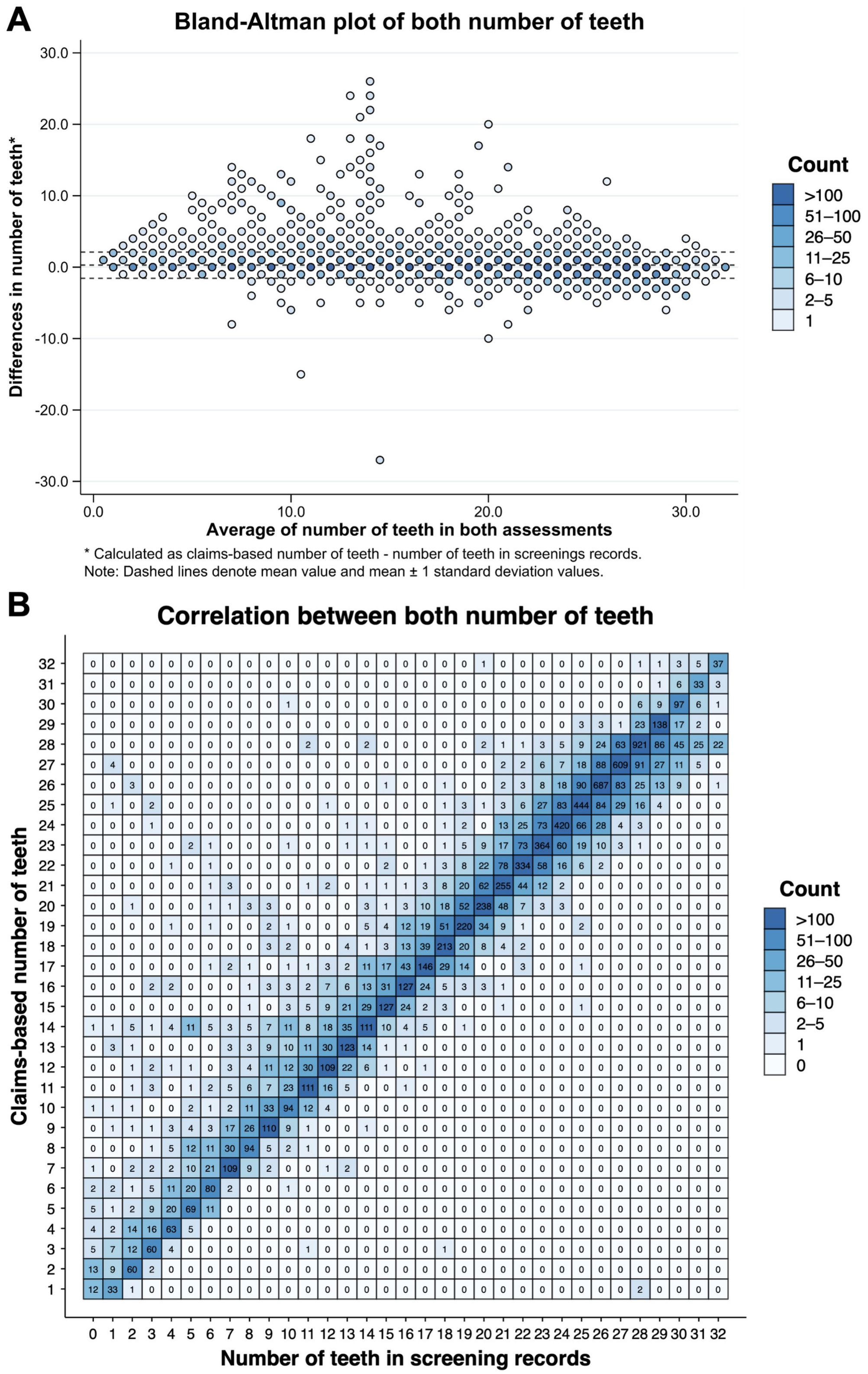

Note: The claims-based number of teeth was defined using the latest claims data in 12 months before the screening month.
