# Supplementary material for: Validity of claims-based definition of number of remaining teeth in Japan: Results from the Longevity Improvement and Fair Evidence Study
Source: PLoS One. 2024 May 7;19(5):e0299849. doi: 10.1371/journal.pone.0299849 (PMC11075880; doi:10.1371/journal.pone.0299849)
Supplement: S5 Fig — (PDF) [file pone.0299849.s005.pdf]

Figure S5. Cumulative incidence curves of Alzheimer's disease according to the claims-based number of remaining teeth using Alzheimer's disease definition that integrates Alzheimer's disease medications.

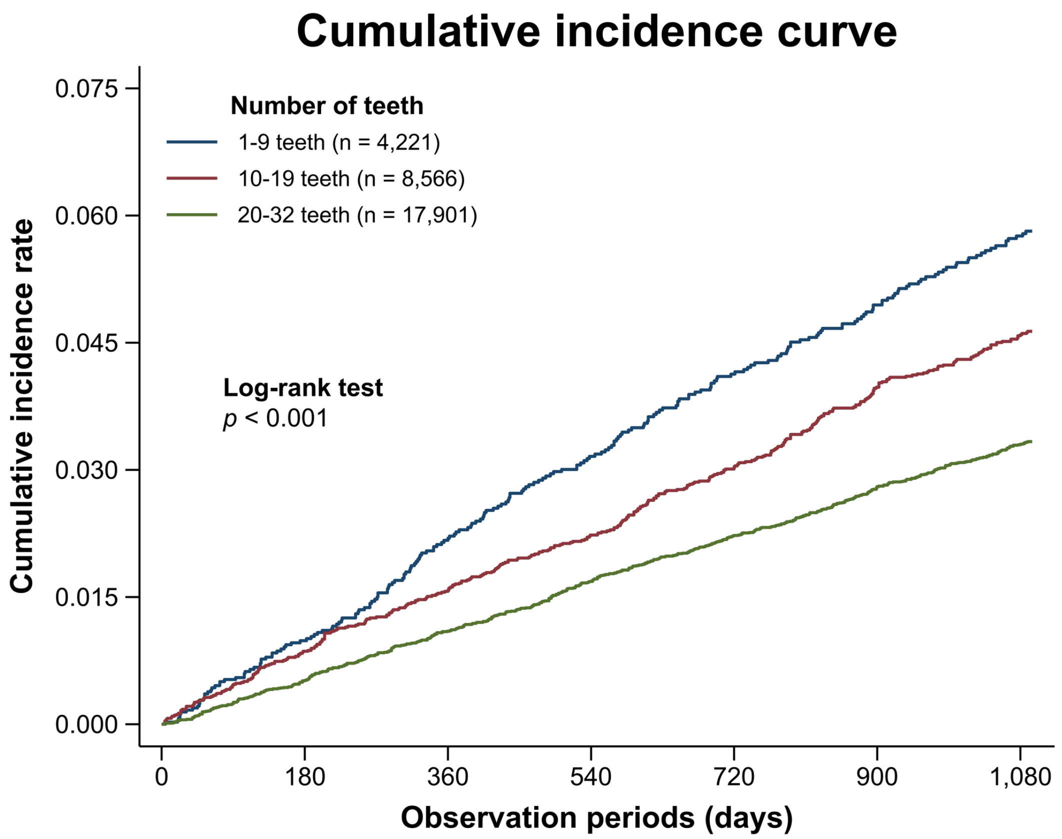

Number at risk

1-9 teeth	4,221	4,070	3,908	3,758	3,583	3,447	3,301
10-19 teeth	8,566	8,351	8,097	7,875	7,623	7,375	7,150
20-32 teeth	17,901	17,537	17,143	16,792	16,440	16,088	15,725
