# Supplementary material for: Validity of claims-based definition of number of remaining teeth in Japan: Results from the Longevity Improvement and Fair Evidence Study
Source: PLoS One. 2024 May 7;19(5):e0299849. doi: 10.1371/journal.pone.0299849 (PMC11075880; doi:10.1371/journal.pone.0299849)
Supplement: S1 Table — (PDF) [file pone.0299849.s006.pdf]

Table S1. Definition of Japanese original diagnosis codes for pneumococcal disease.

	Japanese original diagnosis codes
Pneumococcal disease	8847765, 3201001, 8838800, 7907001, 8847809, 0389004, 8831417, 3229007, 3239028, 8838820, 8838821, 8838818, 8838823, 0389014, 0389015, 0389016, 7907001, 8838819, 8847009, 8838802, 8838798, 4829003, 8832171, 4860030
