# Supplementary material for: Validity of claims-based definition of number of remaining teeth in Japan: Results from the Longevity Improvement and Fair Evidence Study
Source: PLoS One. 2024 May 7;19(5):e0299849. doi: 10.1371/journal.pone.0299849 (PMC11075880; doi:10.1371/journal.pone.0299849)
Supplement: S2 Table — (PDF) [file pone.0299849.s007.pdf]

Table S2. Correlation between claims-based number of teeth and number of teeth in screening records by age group and number of teeth group in screening records.

	Intraclass correlation coefficient
Age group	
20–39 years	0.58
40–64 years	0.97
65–74 years	0.98
≥75 years	0.97
Number of teeth in screening records	
≤9 teeth	0.44
10–19 teeth	0.89
≥20 teeth	0.96
