# Supplementary material for: Validity of claims-based definition of number of remaining teeth in Japan: Results from the Longevity Improvement and Fair Evidence Study
Source: PLoS One. 2024 May 7;19(5):e0299849. doi: 10.1371/journal.pone.0299849 (PMC11075880; doi:10.1371/journal.pone.0299849)
Supplement: S3 Table — (PDF) [file pone.0299849.s008.pdf]

Table S3. Characteristics of analytical sample in prediction analysis for pneumococcal disease according to the claims-based number of remaining teeth.

	Total (n = 30,838)		Number of teeth					
			1–9 teeth (n = 4,333)		10–19 teeth (n = 8,616)		≥20 teeth (n = 17,889)	
	n	%	n	%	n	%	n	%
PD onset								
Yes	3,212	10.4	645	14.9	1,012	11.7	1,555	8.7
Sex								
Male	12,335	40.0	1,724	39.8	3,338	38.7	7,273	40.7
Age group								
65–74 years	10,557	34.2	695	16.0	2,181	25.3	7,681	42.9
75–79 years	8,929	29.0	1,155	26.7	2,548	29.6	5,226	29.2
80–84 years	7,501	24.3	1,377	31.8	2,485	28.8	3,639	20.3
≥85 years	3,851	12.5	1,106	25.5	1,402	16.3	1,343	7.5
Hypertension								
Yes	19,304	62.6	3,019	69.7	5,640	65.5	10,645	59.5
Diabetes								
Yes	11,146	36.1	1,701	39.3	3,313	38.5	6,132	34.3

Abbreviations: PD = pneumococcal disease.
