# Supplementary material for: Validity of claims-based definition of number of remaining teeth in Japan: Results from the Longevity Improvement and Fair Evidence Study
Source: PLoS One. 2024 May 7;19(5):e0299849. doi: 10.1371/journal.pone.0299849 (PMC11075880; doi:10.1371/journal.pone.0299849)
Supplement: S4 Table — (PDF) [file pone.0299849.s009.pdf]

Table S4. Characteristics of analytical sample in prediction analysis for Alzheimer's disease according to the claims-based number of remaining teeth.

	Total (n = 30,207)		Number of teeth					
			1–9 teeth (n = 4,106)		10–19 teeth (n = 8,390)		≥20 teeth (n = 17,711)	
	n	%	n	%	n	%	n	%
AD onset								
Yes	1,589	5.3	324	7.9	509	6.1	756	4.3
Sex								
Male	12,343	40.9	1,715	41.8	3,326	39.6	7,302	41.2
Age group								
65–74 years	10,733	35.5	706	17.2	2,236	26.7	7,791	44.0
75–79 years	8,882	29.4	1,129	27.5	2,530	30.2	5,223	29.5
80–84 years	7,224	23.9	1,301	31.7	2,394	28.5	3,529	19.9
≥85 years	3,368	11.1	970	23.6	1,230	14.7	1,168	6.6
Hypertension								
Yes	18,772	62.1	2,856	69.6	5,456	65.0	10,460	59.1
Diabetes								
Yes	10,964	36.3	1,616	39.4	3,247	38.7	6,101	34.3

Abbreviations: AD = Alzheimer's disease.
