# Supplementary material for: Validity of claims-based definition of number of remaining teeth in Japan: Results from the Longevity Improvement and Fair Evidence Study
Source: PLoS One. 2024 May 7;19(5):e0299849. doi: 10.1371/journal.pone.0299849 (PMC11075880; doi:10.1371/journal.pone.0299849)
Supplement: S5 Table — (PDF) [file pone.0299849.s010.pdf]

Table S5. Association between the claims-based number of remaining teeth and the onset of Alzheimer's disease during follow-up using Alzheimer's disease definition that integrates Alzheimer's disease medications.

	AD (n = 30,688)		
	Person-years at risk	Crude incidence rate†	HR (95% CI‡)§
Number of teeth			
1–9 teeth	11,233.6	20.1	1.08 (0.92–1.26)
10–19 teeth	23,532.3	15.9	1.01 (0.88–1.16)
≥20 teeth	50,328.3	11.3	1.00 (Reference)

Abbreviations: AD = Alzheimer's disease; HR = hazard ratio; CI = confidence interval.

† Per 1,000 person-years.

‡ Obtained by bootstrapping with 1,000 replications.

§ Adjusted for sex, age group, hypertension, and diabetes.
