# Supplementary material for: Validity of claims-based definition of number of remaining teeth in Japan: Results from the Longevity Improvement and Fair Evidence Study
Source: PLoS One. 2024 May 7;19(5):e0299849. doi: 10.1371/journal.pone.0299849 (PMC11075880; doi:10.1371/journal.pone.0299849)
Supplement: S6 Table — (PDF) [file pone.0299849.s011.pdf]

Table S6. Association between the dental status and the onsets of pneumococcal disease and Alzheimer's disease during the follow-up.

	PD (n = 30,838)†			AD (n = 30,207)†		
	Person-years at risk	Crude incidence rate‡	HR (95% CI§)¶	Person-years at risk	Crude incidence rate‡	HR (95% CI§)¶
Dental status						
1–9 teeth not using denture	2,807.9	61.6	1.41 (1.21–1.66)	2,698.4	31.5	1.35 (1.08–1.70)
1–9 teeth using denture	8,305.1	56.8	1.24 (1.12–1.38)	8,189.4	29.2	1.14 (0.98–1.32)
10–19 teeth not using denture	8,827.4	44.1	1.19 (1.07–1.33)	8,811.0	23.6	1.04 (0.88–1.23)
10–19 teeth using denture	14,185.7	43.9	1.08 (0.98–1.19)	14,139.0	20.0	1.06 (0.93–1.22)
≥20 teeth	49,193.2	31.6	1.00 (Reference)	49,681.5	15.2	1.00 (Reference)

Abbreviations: PD = pneumococcal disease; AD = Alzheimer's disease; HR = hazard ratio; CI = confidence interval.

† Each outcome was separately included in the analytic models.

‡ Per 1,000 person-years.

§ Obtained by bootstrapping with 1,000 replications.

¶ Adjusted for sex, age group, hypertension, and diabetes.
